# Factors Associated With Use of Telemedicine for Follow-Up of SLE in the COVID-19 Outbreak

**DOI:** 10.3389/fmed.2021.790652

**Published:** 2021-12-13

**Authors:** Ho So, Evelyn Chow, Isaac T. Cheng, Sze-Lok Lau, Tena K. Li, Cheuk-Chun Szeto, Lai-Shan Tam

**Affiliations:** Department of Medicine and Therapeutics, The Chinese University of Hong Kong, Hong Kong, Hong Kong SAR, China

**Keywords:** COVID-19, lupus nephritis, systemic lupus erythematosus, telehealth, telemedicine

## Abstract

**Objective:** To investigate the factors associated with telemedicine (TM) use for follow-up of Systemic Lupus Erythematous (SLE) patients in the COVID-19 pandemic.

**Methods:** This was a single-centered cross-sectional study conducted in Hong Kong. Consecutive patients followed up at the lupus nephritis clinic were contacted for their preference in changing the coming consultation to TM in the form of videoconferencing. The demographic, socioeconomic, and disease data of the first 140 patients opted for TM and 140 control patients preferred to continue standard in-person follow-up were compared.

**Results:** The mean age of all the participants was 45.6 ± 11.8 years, and the disease duration was 15.0 ± 9.2 years. The majority of them were on prednisolone (90.0%) and immunosuppressants (67.1%). The mean SLEDAI-2k was 3.4 ± 2.4, physician global assessment (PGA) was 0.46 ± 0.62 and Systemic Lupus International Collaborating Clinics (SLICC) damage index was 0.97 ± 1.23. A significant proportion of the patients (72.1%) had 1 or more comorbidities. It was found that patients with higher mean PGA (TM: 0.54 ± 0.63 vs. control: 0.38 ± 0.59, *p* = 0.025) and family monthly income > USD 3,800 (TM: 36.4% vs. control: 23.6%; *p* = 0.028) preferred TM, while full-time employees (TM: 40.0% vs. control: 50.7%; *p* = 0.041) preferred in-person follow-up. These predictors remained significant in the multivariate analysis after adjusting for age and gender. No other clinical factors were found to be associated with the preference of TM follow-up.

**Conclusion:** When choosing the mode of care delivery between TM and physical clinic visit for patients with SLE, the physician-assessed disease activity and patient's socio-economic status appeared to be important.

## Introduction

Since coronavirus disease 2019 (COVID-19) was declared a pandemic, the rapidly increasing number of cases and deaths overwhelmed the health care system worldwide. Systemic lupus erythematosus (SLE) is a chronic remitting-relapsing disease that affects multiple organ systems. Patients with SLE are at heightened risk of infection due to the underlying disease and the use of immunosuppressive therapies (1). The increased prevalence of comorbidities, such as hypertension and cardiovascular diseases, have been reported to be poor prognostic factors of COVID-19 (2, 3). During this extraordinary time, lupus patients face the difficult choice between risking COVID-19 exposure during a clinician visit and postponing needed care. Patients with SLE typically require regular follow-up (FU) visits to ensure early detection of flares and to monitor the toxicity of immunosuppressive therapy. The unattended patients are at risk of sub-optimal disease control which will lead to damage accrual and high costs (4, 5). An alternative option would be to adopt telemedicine (TM) or telehealth, the use of telecommunication technologies to provide medical information and services. In fact, the use of TM to reduce potential exposure to COVID-19 has been recommended by international rheumatology societies (6, 7). Communication via telephone or video consultations are recommended over emails because of privacy concerns (8). Specific statements on the scope and limitations of the use of video consultations in rheumatology patients have also been published (9).

Despite being widely used during the pandemic, evidence of TM in rheumatology is sparse. According to a systematic review in 2017, there is no good evidence in supporting the use of TM for the management of rheumatic diseases (10). In total, 2 studies done during the COVID-19 outbreak reported moderate acceptance of TM as the mode of care in patients with rheumatic diseases (11, 12). However, there is no data on the clinical factors associated with the use of TM in patients with SLE. We hypothesized that the decision of choosing TM as the mode of FU could be predicted by certain patient profile.

In this study, we aimed to examine the demographic, socio-economic, psychological, disease, and treatment factors associated with the patient's preference of use of TM for FU of SLE.

## Methods

### Study Design and Patients

This was a single-center, observational, cross-sectional study. The study was performed at the lupus nephritis clinic of a regional hospital in Hong Kong where most of the patients reside nearby. From May 1 to November 30, 2020, all consecutive adult patients with SLE, according to the 2019 EULAR/ACR classification criteria, were invited to participate in the study (13). Patients (or their carers) needed to possess the technology required to conduct a TM visit (a smartphone, tablet or computer with audio and video capabilities and internet connection) via a real-time video conferencing software ZOOM (Zoom Video Communications Inc, California, US). Patients on intravenous cyclophosphamide were excluded. All patients who had given written informed consent were asked for their interest in changing the coming scheduled FU to TM-based in the form of a videoconference. The first 140 patients agreed to use TM care were recruited. Another 140 consecutive patients who preferred to continue standard FU were enrolled as controls. All participants were asked to complete a set of questionnaires including the LupusQoL, Health Assessment Questionnaire Disability Index (HAQ-DI), and Hospital Anxiety and Depression Scale (HADS). The LupusQoL (0–100, 100 worst) is a disease-targeted patient reported outcome measure that was developed and validated for SLE patients (14). It consists of both 8 health-related and 4 non–health related domains to enable an understanding of the broader burden of the disease. HAQ-DI (0–3, 3 most disabled) covers various common daily activities to assess disability (15). HADS (>8 denotes anxiety or depression) was used to assess anxiety and depression in medical patients (16). The socio-economic status of the patients was also collected through questionnaire which has been used in studies of local patients with autoimmune rheumatic diseases (17, 18). The study was approved by the local research ethics committee (The Joint Chinese University of Hong Kong – New Territories East Cluster Clinical Research Ethics Committee, No. 2020-0254) and conducted according to the principles of the Declaration of Helsinki.

### Assessments

The disease variables recorded included disease duration, comorbidities, nephritis class, ever presence of rash/ joint pain, proteinuria, medications, disease activity, and Systemic Lupus International Collaborating Clinics/American College of Rheumatology (SLICC/ACR) Damage Index (SDI) (19). SLE disease activity was assessed by the Systemic Lupus Erythematosus Disease Activity Index 2000 (SLEDAI-2k) and physician global assessment [PGA (0–3, 3 most active)] (19). Disease remission was defined as absence of clinical activity with no use of systemic glucocorticoids (GC) and immunosuppressive agents; and lupus low disease activity state (LLDAS) as a SLEDAI 2k ≤ 4, PGA ≤ 1 with GC ≤ 7.5 mg of prednisone daily and well tolerated standard maintenance doses of immunosuppressive agents (20). All investigations and assessments were performed within 1 month before or after the patients were recruited. The clinical assessments of the control group were done face-to-face while those for the TM group were by either face-to-face or videoconferencing. All patients were required to come to the hospital for blood and urine tests prior to the scheduled FU. The clinical data were retrieved from the electronic health record (EHR) manually and were documented into a computer database for analysis.

### Statistical Analysis

The overall demographic and clinical characteristics of the recruited patients were reported as mean values with standard deviations for continuous variables and as numbers and percentages for categorical variables. The patients in the TM and control groups were compared by chi-square test or fisher exact test and student *t*-test where appropriate. Binary logistic regression was conducted for analysis of independent predictors with respect to preference over TM FU. Age, gender, and other predictors with *p* < 0.1 in the univariate analyses were put into the regression model. A 2-tailed probability value of *p* < 0.05 was considered statistically significant. Statistical analyses were performed using the Statistics Package for Social Sciences V.26.0 (IBM Corporation, Armonk, NY, USA).

## Results

A total of 332 patients with SLE were screened and 34 were excluded due to the lack of required equipment. The demographic and disease characteristics of the first 140 patients preferred TM and 140 controls are presented in Table 1. The mean age of the 280 recruited patients was 45.6 ± 11.8 years. There was a female predominance (91.4%). The mean disease duration was 15.0 ± 9.2 years. The majority (88.2%) had biopsy proven lupus nephritis class III, IV, or V. The mean SLEDAI-2k was 3.4 ± 2.4, mean PGA was 0.46 ± 0.62 and SDI was 0.97 ± 1.23. Almost all of them (90%) were on prednisolone with a mean daily dose of 5.8 ± 6.1 mg. In total, 2 third of the patients (67.1%) were on immunosuppressive agents with the most common being mycophenolate mofetil followed by calcineurin inhibitors. While 70% of the patients were in LLDAS, only 5 (1.8%) had disease remission. A significant proportion of the patients (72.1%) had 1 or more comorbidities. The mean HAQ-DI was 0.20 ± 0.40. Regarding the anxiety and depression scales, 32.9% and 29.6% of the patients had score equal to or greater than 8, respectively. The socio-economic profile of the patients is presented in Table 2.

**Table 1:** Disease data of the recruited patients and comparison between the telemedicine/standard follow-up groups.

	**Overall** **(***n =*** 280)**	**Telemedicine group** **(***n =*** 140)**	**Standard follow-up group** **(***n =*** 140)**	***P*** **-value**
Age in years	45.6 ± 11.8	44.6 ± 11.4	46.6 ± 12.1	0.159
Gender: Female	256 (91.4)	127 (90.7)	129 (92.1)	0.669
Ever presence of: Rash Joint pain	170 (60.8) 174 (62.1)	87 (62.1) 92 (65.7)	82 (58.6) 82 (58.6)	0.527 0.247
Disease duration in years	15.8 ± 9.5	15.0 ± 9.3	16.5 ± 9.6	0.176
Nephritis class III, IV or V	247 (88.2)	122 (87.1)	125 (89.3)	0.662
24 hour urine proteinuria in gram	0.45 ± 0.60	0.50 ± 0.63	0.40 ± 0.57	0.176
Current use of prednisolone	252 (90.0)	125 (85.7)	127 (90.7)	0.690
Daily prednisolone dose in mg	5.8 ± 6.1	5.3 ± 4.5	6.4 ± 7.4	0.143
Use of immunosuppressant: Mycophenolate mofetil Calcineurin inhibitors Azathioprine Methotrexate Rituximab	188 (67.1) 105 (37.5) 49 (17.5) 28 (10) 3 (1.1) 3 (1.1)	96 (68.6) 62 (44.3) 20 (14.3) 12 (8.6) 1 (0.7) 1 (0.7)	92 (65.7) 43 (30.7) 29 (20.7) 16 (11.4) 2 (1.4) 2 (1.4)	0.611
SLEDAI-2K	3.4 ± 2.4	3.5 ± 2.3	3.3 ± 2.4	0.366
PGA	0.46 ± 0.62	0.54 ± 0.63	0.38 ± 0.59	**0.025**
LLDAS	196 (70)	92 (65.7)	104 (74.3)	0.160
Remission	5 (1.8)	0 (0)	5 (3.6)	0.060
Presence of comorbidity	202 (72.1)	100 (71.4)	102 (72.9)	0.790
Number of comorbidity	1.5 ± 1.4	1.4 ± 1.4	1.6 ± 1.4	0.301
SDI	0.97 ± 1.23	0.95 ± 1.21	1.00 ± 1.26	0.732
HAQ-DI	0.20 ± 0.40	0.23 ± 0.45	0.18 ± 0.34	0.300
HADS: Anxiety scale Depression scale	5.9 ± 4.0 5.6 ± 3.9	5.9 ± 4.1 5.6 ± 4.3	6.0 ± 3.9 5.6 ± 3.5	0.776 0.954
LupusQoL score for: Physical health Pain Planning Intimate relationship Burden to others Emotional health Body image Fatigue	80.5 ± 18.8 81.3 ± 18.8 83.8 ± 18.7 77.1 ± 24.9 73.9 ± 23.2 81.2 ± 17.4 78.8 ± 22.1 74.2 ± 20.6	79.6 ± 20.2 82.1 ± 19.2 83.8 ± 18.7 74.7 ± 27.6 75.4 ± 22.3 82.1 ± 17.5 78.4 ± 23.2 75.6 ± 19.9	81.5 ± 17.1 80.4 ± 18.4 83.8 ± 18.8 79.7 ± 21.3 72.4 ± 24.1 80.2 ± 17.3 79.1 ± 21.0 72.7 ± 21.3	0.406 0.456 0.996 0.201 0.295 0.383 0.793 0.247

*Data are reported as mean ± SD or number (%). SLEDAI-2k, Systemic Lupus Erythematosus Disease Activity Index 2000; PGA, physician global assessment; LLDAS, lupus low disease activity state; SDI, Systemic Lupus International Collaborating Clinics/American College of Rheumatology (SLICC/ACR) Damage Index; HAQ-DI, Health Assessment Questionnaire Disability Index; and HADS, Hospital Anxiety and Depression Scale*.

**Table 2:** Socio-economic data of the recruited patients and comparison between the telemedicine/standard follow-up groups.

	**Overall** **(***n =*** 280)**	**Telemedicine group** **(***n =*** 140)**	**Standard follow-up group** **(***n =*** 140)**	***P*** **-value**
Sick leave due to SLE in the past year	91 (32.5)	44 (31.4)	47 (33.6)	0.929
Days of sick leave due to SLE in the past year	12.0 ± 19.0	11.2 ± 20.4	12.8 ± 17.9	0.702
Hospitalization due to SLE in the past year	64 (22.9)	36 (25.7)	28 (20.0)	0.313
Days of hospitalization due to SLE in the past year	20.9 ± 24.1	22.6 ± 26.7	18.7 ± 20.5	0.525
Currently married	148 (52.9)	81 (57.9)	67 (47.9)	0.134
Highest education level: No formal education Primary school Secondary school Vocational school Tertiary education	8 (2.9) 15 (5.4) 135 (48.2) 43 (15.4) 79 (28.2)	2 (1.4) 5 (3.6) 70 (50.0) 20 (14.3) 43 (30.7)	6 (4.3) 10 (7.1) 65 (46.4) 23 (16.4) 36 (25.7)	0.322
Fulltime employment	127 (45.4)	56 (40.0)	71 (50.7)	**0.041**
Occupation: Professionals Housewives Students	36 (12.9) 42 (15.0) 11 (3.9)	22 (15.7) 22 (15.7) 7(5.0)	14 (10.0) 20 (14.3) 4 (2.9)	0.181 0.816 0.382
Housing: Private Public	173 (61.8) 107 (38.2)	91 (65.0) 49 (35.0)	82 (58.6) 58 (41.4)	0.325
Distance from home to hospital in KM	6.3 ± 6.0	6.3 ± 6.4	6.4 ± 5.5	0.951
Monthly family income > USD 3,800	84 (30.0)	51 (36.4)	33 (23.6)	**0.028**

*Data are reported as mean ± SD or number (%). SLE, systemic lupus erythematosus*.

Univariate analyses showed that higher PGA (TM: mean 0.54± 0.63 vs. control: 0.38 ± 0.59, *p* = 0.025) and family monthly income > USD3, 800 (HKD30, 000) (TM: 51/140, 36.4% vs. control: 33/140, 23.6%; *p* = 0.028) were associated with the preference of TM use, while fulltime employment (TM: 56/140, 40.0% vs. control: 71/140, 50.7%; *p* = 0.041) was related to physical FU. There was no statistically significant difference in the objective parameters of disease activity. There was also no other difference in the demographics, socio-psychological factors, lupus manifestations, disease damage, co-morbidities, and pharmacotherapies between the 2 groups of patients.

Binary logistic regression analysis revealed higher PGA, family monthly income > USD3, 800, and non-fulltime employment status remained independently associated with TM care (OR 1.05 95% CI 1.01–1.09 *p* = 0.027, OR 1.90 95% CI 1.24–3.79 *p* = 0.007, OR 1.89 95% CI 1.13–3.17 *p* = 0.015, respectively) after adjustment for age and gender.

## Discussion

As we define the new normal for ambulatory care in the COVID era, we need a new approach to provide FU for our SLE patients, and TM is an obvious option. While there was conflicting evidence about the effectiveness of TM in an early systematic review, subsequent individual studies on specific disease entities have been promising (21). When compared with in-person care, TM resulted in greater reductions of severity in patients with depression, equally reliable outcome assessment in patients with low back pain, and similarly improved skin score in patients with psoriasis (22–24). It was also suggested that TM helped to balance the healthcare workforce and to address manpower insufficiency (25, 26). A randomized controlled trial in 2018 found that a TM FU could achieve similar disease control as conventional care in rheumatoid arthritis patients with low disease activity or remission (27). The COVID-19 pandemic compelled the rapid adoption of TM in rheumatology. For instance, 1 Italian study reported a smooth switch of 80% of the outpatient appointments to TM (28). Another study done in the US noted that TM peaked at 92% of the total visits and was accompanied by a large shift in provider EHR utilization (29). A research letter reported that 52.7% of patients with predominantly arthritis in a rheumatology department in Spain considered phone consultation to be useful, and no specific patient profile was associated with this opinion (30). It was also commented by the authors of a study reporting the experience of a rheumatology teleclinic in the UK that old age or presence of comorbidities were not reasons for not offering TM FU (31). However, data relate specifically to patients with SLE are scarce.

It might appear intuitive that TM is more suitable and easily accepted by patients with milder disease. On the other hand, the benefit of offering TM FU in patients with major organ involvement who might just omit FU for the fear of COVID-19 infection would be more pronounced. In a representative population of patients with significant lupus disease mostly requiring systemic glucocorticoid and immunosuppressive agents, we found that higher physician-assessed disease activity was associated with the preference of TM FU. This could be due to the fear of infection exposure during clinic visits in patients with more active disease, as we have previously found that choice of TM FU was associated with the perception that TM FU would reduce the risk of infection while routine care would increase that risk (10). In fact, a survey distributed to patients with SLE during the outbreak showed that their median fear of COVID-19 was 8 out of a maximum scale of 10 (32). Interestingly, in a study done before the COVID-19 outbreak, when offered as an option, video TM was also more likely to be used by rheumatoid arthritis patients with higher disease activity (33). Another possible explanation for the higher PGA in the TM group could be the perceived less stable disease when the patients were assessed virtually. It should also be noted that the small difference in PGA might not be clinically meaningful. Other clinical factors such as lupus manifestations, objective activity, disease damage, pharmacotherapies, disability, and depression/anxiety symptoms did not seem to affect patients' choice of mode of FU.

In this study, we also found that higher monthly family income favored TM use. Cavagna et al. reported the results of a survey on the propensity for adopting TM in 175 patients with connective tissue disease of whom 49 had SLE (11). It was found that a college degree and distance from the hospital were independent predictors for the acceptance of TM. It might seem conceivable that patients who are socio-economically more privileged would be keener to use TM (34). The issue needs to be addressed before universal integration between TM and standard care in order not to exacerbate health care disparities. On the other hand, we found no association in the distance from hospital with the preference of TM. This could be related to the fact that most of our patients were residing close to the hospital.

Another intriguing finding of the study is the association of fulltime employment status with standard in-person visit. Complete society lock-down or prohibition of social mobility was not in place in Hong Kong which meant patients with fulltime employment still had to go to work. As a result, the increased infection risk associated with attending the scheduled clinic FU might seem to be negligible.

There are several limitations in this study. First, the results should be interpreted in the context of the local outbreak status and mitigation measures implemented. Second, the study was conducted in a lupus nephritis clinic with mainly Asian patients having major organ involvement. The results might not be generalizable to the entire SLE population, which would include more patients with mild disease. Lastly, the suitability, mainly reflected by safety and efficacy, of TM FU in patients with SLE was not evaluated in the current study.

To conclude, when offered as an option in SLE patients, preference for TM FU was associated with non-fulltime employment, higher physician-determined disease activity, and better family income. With the availability of vaccines and gradual loosening of containment measures, the results could provide information on the factors to consider when we choose the mode of care delivery during and after the COVID-19 outbreak. The physician global assessment and socio-economic status, rather than other clinical factors such as treatments or comorbidities, appeared to be the important determinants of mode of follow-up in lupus patients.

## Data Availability Statement

The raw data supporting the conclusions of this article will be made available by the authors, without undue reservation.

## Ethics Statement

The studies involving human participants were reviewed and approved by the Joint Chinese University of Hong Kong – New Territories East Cluster Clinical Research Ethics Committee, No. 2020-0254. The patients/participants provided their written informed consent to participate in this study.

## Author Contributions

HS, C-CS, and L-ST: study design. HS, EC, IC, S-LL, and TL: data collection and data analysis. HS and L-ST: drafting of manuscript. All authors critically revised the manuscript for important intellectual content.

## Conflict of Interest

The authors declare that the research was conducted in the absence of any commercial or financial relationships that could be construed as a potential conflict of interest.

## Publisher's Note

All claims expressed in this article are solely those of the authors and do not necessarily represent those of their affiliated organizations, or those of the publisher, the editors and the reviewers. Any product that may be evaluated in this article, or claim that may be made by its manufacturer, is not guaranteed or endorsed by the publisher.
